# The association of high school male teachers’ physical education classroom environment with academic buoyancy among female students: a chain mediating role of positive academic emotions and learning engagement

**DOI:** 10.3389/fpsyg.2026.1897866

**Published:** 2026-09-29

**Authors:** Xiaojun Song, Xiangzheng Meng

**Affiliations:** 1 School of General Education, Liaoning University of International Business and Economics, Dalian, China; 2 School of Physical Education, Liaoning Normal University, Dalian, China

**Keywords:** academic buoyancy, female high school students, learning engagement, physical education classroom environment, positive academic emotions

## Abstract

**Introduction:**

This study aimed to examine the associations of high school male teachers’ physical education classroom environment with female students’ academic buoyancy, and to examine the chain mediating role of positive academic emotions and learning engagement.

**Methods:**

Using the Perceived Physical Education Classroom Environment Scale, the Academic Buoyancy Scale, the Academic Emotions Scale, and the Learning Engagement Scale, a survey was conducted among 630 female high school students from five high schools in Northeast China, yielding 601 valid questionnaires. Data were analyzed using Pearson correlation, multiple regression, and mediation analysis with bootstrapping (PROCESS Model 6).

**Results:**

(1) High school male teachers’ physical education classroom environment was significantly positively correlated with female students’ academic buoyancy (*r* = 0.363, *p* < 0.01), positive academic emotions (*r* = 0.274, *p* < 0.01), and learning engagement (*r* = 0.298, *p* < 0.01); Positive academic emotions were significantly positively correlated with learning engagement (*r* = 0.314, *p* < 0.01) and academic buoyancy (*r* = 0.341, *p* < 0.01); Learning engagement was significantly positively correlated with academic buoyancy (*r* = 0.313, *p* < 0.01). (2) Physical education classroom environment was positively and significantly associated with female students’ academic buoyancy, with a direct association of 0.2525, accounting for 69.60% of the total effect. (3) In the relationship between physical education classroom environment and academic buoyancy, positive academic emotions and learning engagement showed both independent mediating effects and a chain mediating effect, with effect sizes accounting for 16.50, 10.70, and 3.20% of the total effect, respectively.

**Conclusion:**

High school male teachers’ physical education classroom environment is positively correlated with female students’ academic buoyancy, and shows a positive indirect association not only through the independent mediating roles of positive academic emotions and learning engagement but also through their chain mediating role. Given the cross-sectional self-report design, these findings should be interpreted as correlational evidence rather than causal conclusions.

## 1 Introduction

Academic buoyancy refers to students’ ability to positively adapt to and successfully cope with everyday academic setbacks and challenges. Different from academic resilience, academic buoyancy focuses more on students’ capacity to handle typical academic obstacles such as poor performance and homework pressure in daily learning contexts (Kritikou and Giovazolias, 2022). Academic buoyancy is influenced by multiple factors (Putwain et al., 2015), and significant gender differences exist among high school students, with male students reporting significantly higher levels of academic buoyancy than female students (Chen, 2024). High school is a critical transitional period for female students’ physical and psychological development. In physical education classes during this stage, female high school students exhibit certain psychological and behavioral particularities. Some research suggests that female high school students may exhibit certain psychological and behavioral particularities in PE classes, including a tendency to prefer quietness over physical activity, resistance to practicing certain movement ranges, and greater vulnerability to self-esteem and psychological adaptation issues in courses that require body image expression (Keh et al., 2013). However, these patterns are not universal and may vary across individuals and contexts. Existing research indicates that male teachers account for approximately half of the high school PE teacher workforce, and teachers’ gender may be associated with differences in teaching styles and evaluation orientations (Huang, 2021). However, the specific effects of teacher gender on classroom dynamics are complex and context-dependent, and disentangling gender-specific effects from other teaching characteristics (e.g., pedagogical approach, classroom management style) requires comparative designs that are beyond the scope of the present study. Nevertheless, examining female students’ experiences in classroom settings led by male teachers is empirically and practically relevant, given that a substantial proportion of high school PE classes are taught by male teachers. In a physical education classroom environment dominated by high school male teachers, female students may encounter particular classroom dynamics and learning experiences that warrant empirical investigation. While our design does not permit attributing observed associations specifically to teacher gender, understanding these classroom dynamics in the male-teacher context is a necessary first step for generating hypotheses that can be tested in future comparative research. The physical education classroom environment may influence female high school students’ academic buoyancy through multiple pathways. A supportive classroom atmosphere first helps evoke positive emotional experiences such as joy and pride during the learning process; such positive affective states can further translate into engagement behaviors, including concentration, effort, and persistence in classroom activities (Zhong, 2024). Moreover, students’ daily academic resilience is closely related to their emotional experiences in learning (Putwain and Daly, 2013). Hence, in-depth investigation into how the physical education classroom environment is associated with female high school students’ academic buoyancy through positive academic emotions and learning engagement is crucial for promoting the physical and psychological health development of this group, expanding the application boundaries of these constructs in physical education contexts, and providing new empirical evidence for interventions targeting female high school students’ mental health.

## 2 Research hypothesis

### 2.1 The proposed positive association between high school male teachers’ physical education classroom environment and academic buoyancy among female students

Physical education classroom environment refers to a multidimensional and multifactorial environmental system that constrains and controls the occurrence and development of physical education teaching, typically comprising internal environment (e.g., teachers’ mood, students’ emotions, selection of teaching materials, teaching methods) and external environment (e.g., facility layout, equipment preparation, surrounding conditions, and hygiene conditions) (Zhang, 2004). For the purpose of this study, physical education classroom environment specifically refers to the learning environment system formed by factors such as classroom atmosphere, teacher-student interaction patterns, and teaching evaluation orientation within the physical education teaching context in which the teachers are male. It is important to emphasize that teacher gender in this study is a characteristic of the research context rather than an experimentally manipulated independent variable. We acknowledge that the observed classroom characteristics may reflect a combination of teaching style, classroom management approach, and contextual factors, and our study design does not permit isolating the unique contribution of teacher gender. The physical education teaching environment has a significant impact on students’ learning activities, and a supportive classroom atmosphere can effectively stimulate students’ willingness to participate and promote their physical and mental health development (Kern and Patton, 2024). When the physical education classroom environment meets students’ basic psychological needs, students are more likely to develop interest in physical education learning, and this interest further translates into sustained learning behaviors (Liu et al., 2025). Moreover, by creating a supportive classroom environment, physical education teachers can provide emotional support for students facing academic difficulties, thereby potentially helping them actively cope with daily academic setbacks and challenges (Richards et al., 2020). Based on this, we propose Hypothesis H1: High school male teachers’ physical education classroom environment has a positive association with female students’ academic buoyancy.

### 2.2 The proposed mediating role of positive academic emotions

Positive academic emotions refer to the positive emotional states related to academic activities that students experience during the learning process (Tan et al., 2021). In the field of physical education, positive academic emotions play a non-negligible role; every success or failure and the resulting emotions in physical education learning may significantly impact students’ psychological health and subsequent learning (Huang, 2016). This indicates that in the physical education classroom, when students experience higher levels of positive emotions, they are more likely to achieve favorable learning outcomes. Meanwhile, students’ sense of control and value regarding academic tasks constitute the core sources of the generation and development of academic emotions, and environmental factors indirectly affect academic emotions by influencing students’ control-value appraisal outcomes (Yang, 2017). Within this theoretical framework, academic emotions can be classified into four categories based on two dimensions: valence (positive–negative) and arousal level (high arousal–low arousal). Among these, positive high-arousal academic emotions include pride, joy, hope, etc., while positive low-arousal academic emotions include contentment, calmness, relaxation, etc. Research has shown that positive academic emotions play an important facilitative role in students’ academic development, being closely related not only to positive learning outcomes such as school engagement, self-regulated learning, and high academic achievement but also to effectively expanding students’ cognitive resources and stimulating learning motivation (Lei et al., 2016). Based on this, we propose Hypothesis H2: High school male teachers’ physical education classroom environment has a positive association with positive academic emotions, and Positive academic emotions in turn have a positive association with female students’ academic buoyancy, thereby playing a mediating role in the relationship between high school male teachers’ physical education classroom environment and female students’ academic buoyancy.

### 2.3 The proposed mediating role of learning engagement

Learning engagement refers to the comprehensive state in which students exhibit behavioral engagement, emotional engagement, and cognitive engagement in learning activities; these three dimensions are interrelated and interactive, collectively forming the complete structure of learning engagement (Xu et al., 2025). The level of learning engagement in physical education classrooms is not only related to students’ physical health but also significantly affects their academic performance, psychological health, and social adaptability (Tannoubi et al., 2023). Students’ learning engagement is a direct reflection of their adaptation to the instructional environment. Enhancing students’ learning engagement in physical education can not only improve their academic quality but also increase their level of activity in physical education classrooms and promote their physical and psychological health development (Schweder and Raufelder, 2024). Students with higher levels of academic buoyancy tend to participate in learning activities in a more active and proactive manner, demonstrating greater persistence and effort; conversely, insufficient learning engagement may deplete students’ psychological resources for coping with daily academic difficulties and potentially reduce their academic buoyancy levels (Gao, 2025). Furthermore, learning engagement may serve as a driving force for academic buoyancy, continuously consolidating and enhancing students’ resilience to setbacks through sustained positive learning experiences (Ye et al., 2025). The two may exhibit a mutually reinforcing, spiraling dynamic relationship: good academic buoyancy provides a psychological foundation for learning engagement, while sustained learning engagement in turn strengthens an individual’s level of academic buoyancy. While reverse causality is theoretically plausible (i.e., students with higher academic buoyancy may be more likely to engage in learning activities), the present study adopts the direction from learning engagement to academic buoyancy based on conservation of resources theory, which posits that sustained behavioral investment in learning activities helps individuals accumulate psychological capital and coping resources that enhance resilience to setbacks. Additionally, this directional assumption is consistent with the temporal ordering of our theoretical model, in which classroom environment influences engagement, and engagement in turn influences adaptive outcomes. Nevertheless, we acknowledge that the cross-sectional nature of this study precludes testing the reverse direction; future longitudinal research is needed to examine the reciprocal relationship more definitively. Based on this, we propose Hypothesis H3: High school male teachers’ physical education classroom environment has a positive association with learning engagement, and learning engagement in turn has a positive association with female students’ academic buoyancy, thereby playing a mediating role in the relationship between high school male teachers’ physical education classroom environment and female students’ academic buoyancy.

### 2.4 The proposed chain mediating roles of positive academic emotions and learning engagement

Chain mediating effect refers to an indirect pathway in which an independent variable is statistically related to a dependent variable through the sequential transmission via two or more mediating variables. External environmental factors first affect the positive emotional states students experience during the learning process, and positive emotions may in turn further promote engagement in learning behaviors (Fierro-Suero et al., 2023). When students experience positive emotional experiences toward academic activities, their learning motivation and participatory behaviors may be significantly enhanced. Positive emotions can expand individuals’ cognitive resources and stimulate exploratory behaviors, thereby potentially promoting increased learning engagement. Therefore, students with higher levels of positive emotions are more likely to exhibit sustained learning participation behaviors (Yang et al., 2024). Accordingly, positive academic emotions may be an important antecedent of learning engagement—after experiencing positive emotions such as joy and pride in the physical education classroom, these emotional states may further translate into higher levels of behavioral participation, emotional engagement, and cognitive focus (Guo et al., 2025). Based on this, we propose Hypothesis H4: There is a positive association between positive academic emotions and learning engagement, such that they play a chain mediating role in the relationship between high school male teachers’ physical education classroom environment and female students’ academic buoyancy (see Figure 1 for the model diagram).

**Figure 1:**
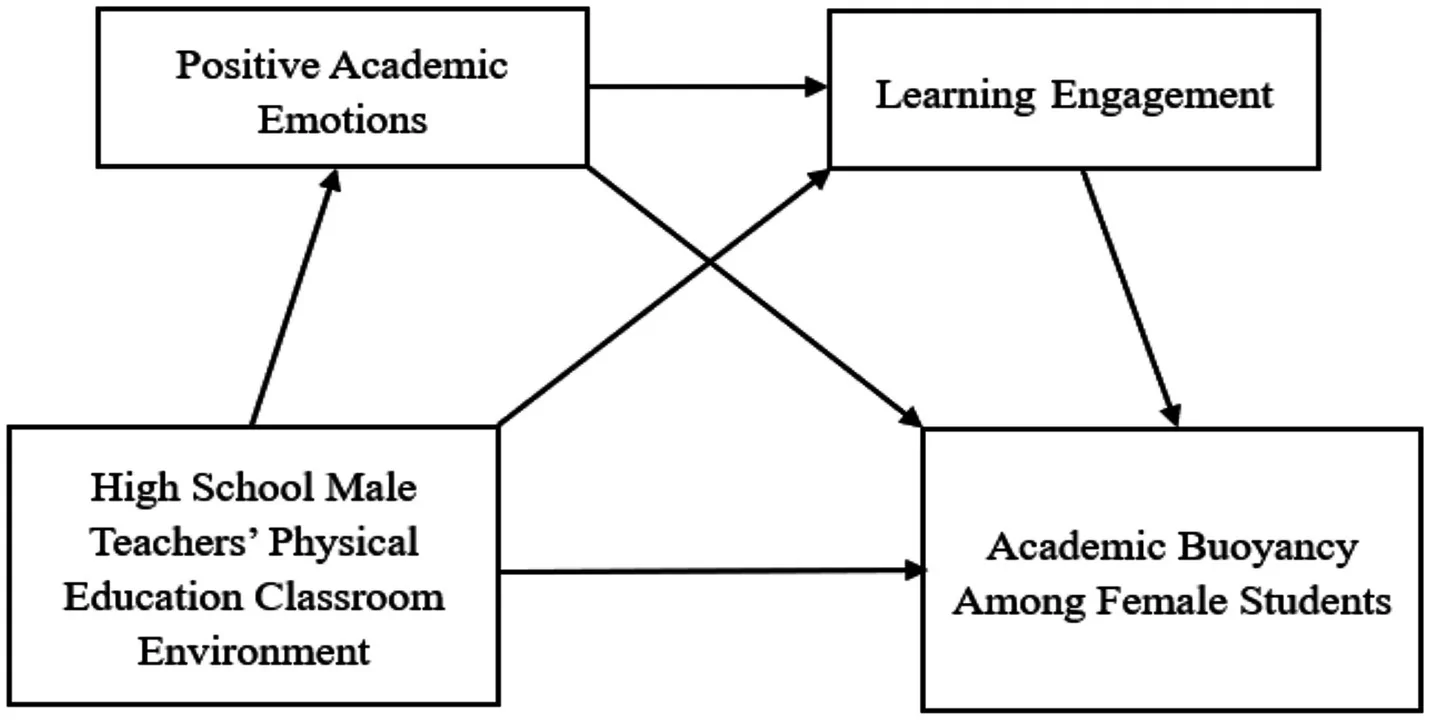
Diagram of the hypothetical model.

## 3 Methodology

### 3.1 Participants and sampling

This study focused on female high school students taught by male physical education teachers in Northeast China. A multistage sampling strategy combining purposive and stratified random sampling was employed to recruit participants.

School selection. Five public high schools were purposively selected from Dalian and its surrounding areas in Liaoning Province. The selected schools comprised two urban schools (located in Dalian city proper) and three suburban schools (in surrounding districts), with student enrollments ranging from approximately 1,200 to 2,500. Two schools were provincial-level key schools, while the remaining three were district-level regular schools. This variation in school type and location was intended to enhance sample representativeness across different educational settings within the region. Selection criteria also included: (a) a comparable number of male and female physical education teachers; (b) complete physical education curricula across all three grades; and (c) willingness of school administrators and departments to participate.

Sampling strata and student selection. Within each school, stratification was conducted by grade level (Senior 1, 2, 3). From each grade, two classes taught by male physical education teachers were randomly selected, yielding 30 classes across the five schools. All female students in these classes were invited to participate. A total of 15 male physical education teachers were represented (one per class), with each teacher responsible for two classes. Teachers who taught across multiple grades were counted once, and their classes were treated as nested within the teacher.

Participant recruitment and data collection. Data collection was conducted between March and April 2026. Prior to distribution, researchers visited each school to explain the study purpose, procedures, and voluntary nature of participation. Questionnaire booklets were distributed at the end of regular physical education classes to avoid disrupting instruction. Students were given approximately 20 min to complete the questionnaires under the supervision of trained research assistants; no physical education teachers were present during administration to minimize social desirability bias.

Eligibility and exclusion criteria. Eligible participants were female students who: (a) were enrolled in Senior 1, 2, or 3 at a selected school; (b) had been taught by a male physical education teacher for at least one full semester prior to data collection; and (c) provided both guardian consent and student assent. Exclusion criteria—established prior to data collection—included: (a) incomplete questionnaires (more than 10% of items missing); (b) patterned responses (selecting the same option for >90% of consecutive items); (c) contradictory responses (inconsistent answers to paired reverse-coded items); and (d) completion time under 5 min (indicating insufficient attention).

Sample size and power. A total of 658 eligible students were invited, of whom 630 agreed to participate (response rate: 95.7%). Of the 630 collected questionnaires, 29 were excluded based on the predetermined criteria: 11 for incomplete responses, 9 for patterned responses, 6 for contradictory responses, and 3 for insufficient completion time. The final analytical sample comprised 601 valid questionnaires (effective response rate: 95.39%). The number of valid participants from each school ranged from 112 to 127, with relatively balanced grade distribution: Senior 1 (*n* = 203, 33.8%), Senior 2 (*n* = 194, 32.3%), and Senior 3 (*n* = 204, 33.9%). Participants’ ages ranged from 15 to 18 years (*M* = 16.42, *SD* = 0.98), consistent with the typical age range for senior high school students in China. According to a Monte Carlo power analysis for mediation models using PROCESS, a sample size of 601 provides sufficient power (>0.95) to detect small-to-medium indirect effects (*α* = 0.05, power = 0.80) in a serial mediation model with two mediators, and exceeds the commonly recommended minimum of 200 participants for structural equation modeling.

Ethical approval. This study was conducted in accordance with the Declaration of Helsinki and approved by the Ethics Committee of Liaoning University of International Business and Economics. Prior to data collection, written informed consent was obtained from the guardians of all participating students, and written assent was obtained from the students themselves. Participants were informed of the study purpose, the voluntary nature of their participation, and their right to withdraw at any time without penalty. All data were anonymized prior to analysis.

### 3.2 Research method

#### 3.2.1 Literature method

A systematic literature review was conducted to construct the theoretical framework and identify research gaps. Relevant literature was identified through searches of databases including CNKI, Web of Science, and PubMed using keywords related to physical education classroom environment, academic buoyancy, academic emotions, and learning engagement.

#### 3.2.2 Questionnaire survey method

Questionnaire survey was used as the primary data collection method. The selected scales (described in detail in Section 2.3) were compiled into a booklet and administered to participants following the stratified random sampling procedure described in Section 2.1.

All scales used in this study were originally developed in English or Chinese. For scales originally developed in English (Academic Buoyancy Scale, Academic Emotions Scale, Learning Engagement Scale), we adopted previously validated Chinese translations from the published literature. For the Perceived Physical Education Classroom Environment Scale, which was originally developed in Chinese, no translation was required. All scales were reviewed by three experts in educational psychology and physical education pedagogy to ensure content relevance and clarity for Chinese high school students. A pilot test was conducted with 30 female high school students (not included in the final sample) to check for comprehension and readability; no significant issues were identified, and minor wording adjustments were made for clarity. Permissions for scale use were obtained from the original developers or through the published scale sources.

Several procedural safeguards were implemented during data collection to minimize the potential influence of common method bias. First, questionnaires were administered anonymously, and participants were assured that their responses would be used solely for research purposes and would not affect their course grades or teacher evaluations. Second, the order of the scales was counterbalanced across participants to reduce order effects. Third, items within each scale were presented in a random order. Fourth, participants were informed that there were no right or wrong answers to reduce evaluation apprehension. Fifth, data were collected from multiple schools and classes rather than from a single site, increasing the representativeness and reducing context-specific biases. These procedural safeguards were implemented in addition to the statistical assessments of common method bias (Harman’s single-factor test, the unmeasured latent method factor approach, and discriminant validity checks) reported in Section 3.1.

#### 3.2.3 Mathematical and statistical method

Data were analyzed using SPSS 29.0. Preliminary analyses included reliability assessment (Cronbach’s alpha), common method bias testing (Harman’s single-factor test and additional checks; see Section 3.1), and descriptive statistics. Pearson correlation analysis and mediation analysis using PROCESS macro for SPSS (Version 4.2, Model 6) with 5,000 bootstrap resamples were conducted to test the hypothesized pathways. All variables in the mediation model were analyzed in their original unstandardized form. All variables were coded such that higher scores indicated higher levels of the respective construct. No missing data were present in the final analytical sample of 601 participants, as all incomplete questionnaires had been excluded during the data cleaning phase. Regression assumptions (linearity, normality of residuals, homoscedasticity, and absence of multicollinearity) were checked prior to the mediation analysis. Variance inflation factor (VIF) values ranged from 1.12 to 1.58, all below the common threshold of 10, indicating no substantial multicollinearity. Residual plots showed no severe violations of homoscedasticity or normality.

To account for the nested structure of participants within schools and classes, we computed intraclass correlation coefficients (ICCs) for the outcome variable (academic buoyancy) at the school and class levels. The ICC values were 0.031 for school and 0.042 for class, indicating that less than 5% of the total variance was attributable to between-school or between-class differences. Previous methodological research suggests that ICC values below 0.05 typically indicate negligible clustering effects, and single-level analyses remain appropriate under such conditions. Given the minimal clustering effects observed, single-level analyses were deemed appropriate. However, we acknowledge this limitation and, in the limitations section (Section 4.7), explicitly discuss the nested structure as a potential concern and recommend that future research with larger samples across more schools consider applying multilevel modeling or cluster-robust standard errors as sensitivity checks.

### 3.3 Research instruments

#### 3.3.1 Perceived physical education classroom environment scale

The Perceived Physical Education Classroom Environment Scale, developed by Tong (2022) based on the theories of Moos and Trickett (1974) and specifically designed for Chinese middle school students, was used to assess students’ perceptions of their physical education classroom environment. The scale comprises 25 items across five dimensions: Teacher Support (5 items, e.g., “My PE teacher encourages me to do my best”), Peer Support (5 items, e.g., “My classmates help me when I have difficulty in PE class”), Task Climate (5 items, e.g., “In PE class, improving my skills is more important than winning”), Performance Climate (5 items, e.g., “In PE class, students are compared with each other”), and Rule Clarity (5 items, e.g., “The rules in PE class are clear to me”). Items are rated on a 5-point Likert scale ranging from 1 (“strongly disagree”) to 5 (“strongly agree”). A total score was used in the analysis, with higher scores indicating a more positive perceived classroom environment. This approach was adopted for several reasons. First, theoretically, the five dimensions are conceptualized as interrelated components of a global classroom environment construct, and their combined score reflects the overall quality of the classroom climate experienced by students. Second, empirically, the dimensions showed moderate to high intercorrelations in the original validation study, supporting their aggregation into a single composite score. Third, for the purpose of the present study, we were interested in the overall perceived classroom environment rather than the unique contribution of individual dimensions, consistent with prior research examining classroom environment as a global predictor of student outcomes. Regarding the inclusion of the Performance Climate dimension (which emphasizes comparison and competition), we acknowledge that this dimension may be perceived as less positive than the other four dimensions; however, its inclusion in the total score reflects the reality that classroom environments typically contain both supportive and pressuring elements. The total score thus represents a holistic assessment of students’ overall experience, balancing the positive and challenging aspects of the classroom environment. The scale demonstrated good construct validity in Chinese middle school samples in the original validation study, with confirmatory factor analysis showing acceptable model fit (CFI = 0.919, RMSEA = 0.058). In the present study, the Cronbach’s alpha coefficient was 0.985, and McDonald’s omega was 0.987. To examine whether the high alpha value reflected potential item redundancy, we conducted a confirmatory factor analysis using the five-factor structure from the original validation study. The model showed acceptable fit: *χ*^2^*/d ~ f ~* = 2.41, CFI = 0.922, RMSEA = 0.056 (90% CI [0.051, 0.061]), SRMR = 0.044, confirming the factor structure in our sample.

#### 3.3.2 Academic buoyancy scale (ABS)

The Academic Buoyancy Scale (ABS), originally developed by Martin and Marsh (2008) and later translated and validated for Chinese secondary students by Sun (2009), was used to assess students’ ability to cope with everyday academic setbacks. The Chinese version adopted in the present study followed the version reported by Zhou (2025). The scale has a unidimensional structure consisting of 4 items (e.g., “I don’t let study stress get on top of me”; “I’m good at dealing with setbacks at school”). Items are rated on a 5-point Likert scale ranging from 1 (“strongly disagree”) to 5 (“strongly agree”). A total score was used, with higher scores indicating greater academic buoyancy. The Chinese version has demonstrated adequate reliability (*α* = 0.78–0.84) and construct validity in Chinese secondary school samples, with confirmatory factor analysis supporting the unidimensional structure. In the present study, the Cronbach’s alpha coefficient was 0.820, and McDonald’s omega was 0.824.

#### 3.3.3 Academic emotions scale

The positive academic emotions subscale was adapted from the Achievement Emotions Questionnaire (AEQ; Pekrun et al., 2002) and the Academic Emotions Scale (Artino and Jones, 2012). The Chinese version used in the present study was adopted from Wei (2022). For this study, we selected the positive academic emotions dimension, which comprises 6 items (e.g., “I enjoy learning new things in PE class”; “I feel proud when I make progress in PE class”). Items are rated on a 5-point Likert scale ranging from 1 (“strongly disagree”) to 5 (“strongly agree”). A total score was used, with higher scores indicating more frequent or intense positive academic emotions. The Chinese version of this scale has demonstrated acceptable reliability (*α* = 0.82–0.88) and construct validity in Chinese student samples, with confirmatory factor analysis supporting the hypothesized factor structure. Importantly, the scale was adapted specifically for the physical education context by replacing the general academic activity references (e.g., “class” → “PE class”), and this adapted version was used in the present study. While the adapted version has been validated in previous research using similar physical education samples, we also provide additional evidence of construct validity for the present sample. As reported in Section 3.1, the average variance extracted (AVE) for this scale was 0.593, exceeding the squared correlations with other constructs, supporting its discriminant validity. In the present study, the Cronbach’s alpha coefficient was 0.831, and McDonald’s omega was 0.836.

#### 3.3.4 Learning engagement scale

The Learning Engagement Scale is the student version of the Utrecht Work Engagement Scale (UWES-S; Schaufeli et al., 2002), translated and adapted for Chinese students by Fang et al. (2008). The Chinese version used in the present study followed Pan (2025). The scale comprises 17 items across three dimensions: Vigor (6 items, e.g., “When I am in PE class, I feel full of energy”), Dedication (5 items, e.g., “I am enthusiastic about PE class”), and Absorption (6 items, e.g., “Time flies when I am in PE class”). Items are rated on a 7-point Likert scale ranging from 1 (“never”) to 7 (“always”). A total score was used, with higher scores indicating greater learning engagement. The Chinese version has demonstrated adequate construct validity in Chinese student samples, with confirmatory factor analysis supporting the three-factor structure. In the present study, the Cronbach’s alpha coefficient was 0.981, and McDonald’s omega was 0.984. To examine whether the high alpha value reflected potential item redundancy, we conducted a confirmatory factor analysis using the three-factor structure from the original validation study. The model showed acceptable fit: *χ*^2^*/d_f_* = 2.28, CFI = 0.915, RMSEA = 0.061 (90% CI [0.055, 0.067]), SRMR = 0.048, confirming the factor structure in our sample.

## 4 Research results

### 4.1 Control and test of common method bias

In this study, to examine the potential influence of common method bias, Harman’s single-factor test was conducted. All scale items were entered into an unrotated exploratory factor analysis. The results showed that there were four factors with eigenvalues greater than 1, indicating that the data were not dominated by a single factor. The variance explained by the largest factor was 32.164%, which is below the critical criterion of 40%, suggesting that common method bias was not a dominant concern in this dataset.

To further examine the potential influence of common method bias, we employed the unmeasured latent method factor approach. We compared a measurement model with all items loading on their respective theoretical factors to a model that additionally included a common method factor with all items loading on it. The results showed that the inclusion of the common method factor did not substantially improve model fit (*ΔCFI* = 0.012, *ΔRMSEA* = 0.008, *ΔSRMR* = 0.006), suggesting that common method variance did not unduly influence our findings.

Additionally, to strengthen the evidence for construct validity, we examined the discriminant validity of the four scales by comparing the average variance extracted (AVE) for each construct with the squared correlations between constructs. For all construct pairs, the AVE values exceeded the corresponding squared correlations, supporting the discriminant validity of the measures. The AVE values were as follows: Physical Education Classroom Environment = 0.612, Academic Buoyancy = 0.578, Positive Academic Emotions = 0.593, and Learning Engagement = 0.604.

Additionally, to supplement the Cronbach’s alpha coefficients reported in Section 2.3, we calculated McDonald’s omega coefficients for each scale to provide a more robust estimate of internal consistency. The omega coefficients were as follows: Perceived Physical Education Classroom Environment (*ω* = 0.987), Academic Buoyancy (*ω* = 0.824), Positive Academic Emotions (*ω* = 0.836), and Learning Engagement (*ω* = 0.984). These values were comparable to the alpha coefficients, indicating strong internal consistency across all measures. As noted in Sections 2.3.1 and 2.3.4, the high coefficients for the classroom environment and learning engagement scales were further examined through confirmatory factor analysis, which confirmed the factor structures in our sample and did not indicate problematic item redundancy.

### 4.2 Descriptive statistics

A total of 601 female high school students were included as participants in this study. Statistical analyses were conducted on their demographic characteristics, including grade level distribution and daily exercise habits. The results are presented in Table 1. Among the participants, there were 203 first-year students (33.8%), 194 s-year students (32.3%), and 204 third-year students (33.9%), indicating a relatively balanced distribution across the three grades. Regarding daily exercise habits, 346 participants (57.6%) reported having exercise habits, while 255 participants (42.4%) reported no exercise habits. The distribution of these demographic characteristics provides a sample background basis for subsequent exploration of the influence of Physical Education Classroom Environment on Female Students’ Academic Buoyancy.

**Table 1:** Demographic analysis.

Attribute	Category	Quantity	Percentage
Regular exercise habits in daily life	With exercise habits	346	57.6%
	Without exercise habits	255	42.4%
Grade level	Senior 1	203	33.8%
	Senior 2	194	32.3%
	Senior 3	204	33.9%

*N* = 601, valid sample size. All participants were female high school students.

Additionally, to explore the influence of having daily exercise habits or not on each variable, an independent samples t-test was conducted on the sample. Participants were divided into two groups according to whether they had exercise habits, and comparisons were made to determine whether there were significant differences in each variable between the different groups. The measurement results are presented in Table 2.

**Table 2:** Independent samples *t*-test.

Variable	Category	*N*	*M* ± SD	*t*	*p*
High School Male Teachers’ Physical Education Classroom Environment	With exercise habits	346	3.124 ± 0.791	0.455	*p* = 0.650
	Without exercise habits	255	3.094 ± 0.828		
Academic Buoyancy Among Female Students	With exercise habits	346	3.870 ± 0.874	1.672	*p* = 0.095
	Without exercise habits	255	3.748 ± 0.896		
Positive Academic Emotions	With exercise habits	346	3.750 ± 0.912	1.109	*p* = 0.268
	Without exercise habits	255	3.665 ± 0.957		
Learning Engagement	With exercise habits	346	5.416 ± 1.503	−0.768	*p* = 0.443
	Without exercise habits	255	5.510 ± 1.440		

Independent samples *t*-test was conducted to compare differences in study variables between groups with and without exercise habits. N = number of participants; *M* ± SD = mean ± standard deviation; t = *t*-statistic; p = significance level (two-tailed). *p* > 0.05 indicates no statistically significant difference.

As shown in Table 2, independent samples t-tests were conducted to compare female students with and without daily exercise habits across all study variables. The results revealed no statistically significant differences between the two groups in perceived physical education classroom environment (*t* = 0.455, *p* = 0.650, Cohen’s *d* = 0.037, 95% CI [−0.120, 0.194]), academic buoyancy (*t* = 1.672, *p* = 0.095, Cohen’s *d* = 0.137, 95% CI [−0.023, 0.297]), positive academic emotions (*t* = 1.109, *p* = 0.268, Cohen’s *d* = 0.091, 95% CI [−0.069, 0.251]), or learning engagement (*t* = −0.768, *p* = 0.443, Cohen’s *d* = −0.063, 95% CI [−0.223, 0.097]). Levene’s tests for equality of variances indicated that the homogeneity of variance assumption was met for all variables (all *p* > 0.05), supporting the use of standard *t*-tests with pooled variances.

The independent samples *t*-test results in Table 2 showed that the differences between the two groups of female students in high school male teachers’ physical education classroom environment, learning engagement, positive academic emotions, and academic buoyancy were not statistically significant (*p* > 0.05). This finding suggests that, in the present sample, daily exercise habits were not associated with differences in the study variables. All participants were female students taught by male teachers; however, because our study did not include a female-teacher comparison group, these results should be interpreted with caution, and further investigation is needed to examine the potential moderating role of contextual factors.

### 4.3 ANOVA single factor analysis

To examine whether there were differences in each variable across different grades, a one-way ANOVA was conducted to analyze the differences among female students in different grades regarding high school male teachers’ physical education classroom environment, academic buoyancy, positive academic emotions, and learning engagement. The results showed that female students in grades one, two, and three did not exhibit statistically significant differences in any of the four variables, as presented in Table 3.

**Table 3:** One-way analysis of ANOVA for female students of different grades.

Relevant variable	Grade	*M ± SD*	*F*	*p*
High School Male Teachers’ Physical Education Classroom Environment	1(203)	3.112 ± 0.819	1.786	*p =* 0.169
	2(194)	3.033 ± 0.853		
	3(204)	3.185 ± 0.742		
Academic Buoyancy Among Female Students	1(203)	3.830 ± 0.868	0.283	*p =* 0.754
	2 (194)	3.780 ± 0.893		
	3 (204)	3.843 ± 0.897		
Positive Academic Emotions	1 (203)	3.749 ± 0.946	1.614	*p =* 0.200
	2 (194)	3.616 ± 0.949		
	3 (204)	3.772 ± 0.897		
Learning Engagement	1 (203)	5.523 ± 1.420	0.577	*p =* 0.562
	2 (194)	5.364 ± 1.560		
	3 (204)	5.477 ± 1.451		

*M* ± SD indicates mean ± standard deviation. *F* is the *F*-statistic for testing the null hypothesis of equal group means. *p* is the *p*-value; *p* < 0.05 suggests statistically significant differences among groups.

One-way ANOVA results (Table 3) showed no statistically significant differences across the three grade levels in any of the four variables: perceived physical education classroom environment (*F* = 1.786, *p* = 0.169, *η*^2^ = 0.006), academic buoyancy (*F* = 0.283, *p* = 0.754, *η*^2^ = 0.001), positive academic emotions (*F* = 1.614, *p* = 0.200, *η*^2^ = 0.005), and learning engagement (*F* = 0.577, *p* = 0.562, *η*^2^ = 0.002). Levene’s tests indicated that the homogeneity of variance assumption was satisfied for all variables (all *p* > 0.05). Given the non-significant omnibus F-tests, post-hoc comparisons were not pursued.

Based on the results in the table, there were no statistically significant differences among female students in the three grades regarding the four variables: high school male teachers’ physical education classroom environment, academic buoyancy, positive academic emotions, and learning engagement. This finding is inconsistent with the conventional assumption that “as grade level increases and academic pressure rises, students show significant changes in related variables,” and is a phenomenon worth noting. However, because the study employed a cross-sectional design rather than a longitudinal follow-up of the same students, the non-significant grade differences should not be interpreted as evidence of stability over time. Rather, these results suggest that, in this sample, the four study variables did not differ significantly across grade levels at the time of data collection.

### 4.4 Correlation analysis between variables

To examine the strength of the associations among the variables, this study used SPSS software to conduct a Pearson correlation analysis among high school male teachers’ physical education classroom environment, academic buoyancy, positive academic emotions, and learning engagement. The calculated Pearson correlation coefficients showed significant positive correlations among all four variables, providing an important relational basis for subsequently constructing a chain mediation model. The results are presented in Table 4.

**Table 4:** Pearson correlation analysis.

Relevant variable	High school male teachers’ physical education classroom environment	Academic buoyancy among female students	Positive academic emotions	Learning engagement
High School Male Teachers’ Physical Education Classroom Environment	1			
Academic Buoyancy Among Female Students	0.363**	1		
Positive Academic Emotions	0.274**	0.341**	1	
Learning Engagement	0.298**	0.313**	0.314**	1

Significant correlation at the 0.01 level (two-tailed), **p<0.01. The 95% bootstrap confidence intervals for the correlations (based on 5,000 resamples) were: classroom environment–academic buoyancy [0.287, 0.436], classroom environment–positive academic emotions [0.195, 0.350], classroom environment–learning engagement [0.223, 0.371], positive academic emotions–learning engagement [0.235, 0.390], positive academic emotions–academic buoyancy [0.267, 0.413], and learning engagement–academic buoyancy [0.239, 0.385].

As shown in Table 5, Model 6 of the SPSS PROCESS macro developed by Hayes was used to test the chain mediating effect. Consistent with prior theoretical frameworks suggesting that demographic variables such as grade level and physical activity habits may influence students’ academic emotions and engagement, we initially considered including grade level and daily exercise habits as covariates in our mediation model. However, preliminary analyses showed no statistically significant associations between these variables and the primary study variables (*p* > 0.05), and their inclusion did not significantly improve model fit or alter the significance or direction of the hypothesized pathways. Therefore, to maintain model parsimony, we excluded them from the final mediation analysis. This decision is consistent with established practices in mediation modeling, where covariates are retained only when they demonstrate substantive theoretical relevance or empirical association with the outcome variables. The data results indicated that significant positive associations existed among the four dimensions: high school male teachers’ physical education classroom environment, female students’ academic buoyancy, positive academic emotions, and learning engagement.

**Table 5:** Regression analysis between variables.

Regression equation		Overall fit index			Significance of regression coefficients		
Outcome variable	Predictor variable	*R*	*R* ^2^	*F*	*β*	*t*	*p*
Positive Academic Emotions (M1)	High School Male Teachers’ Physical Education Classroom Environment (X)	0.274	0.075	48.771	0.274	6.984	<0.001
Learning Engagement (M2)	High School Male Teachers’ Physical Education Classroom Environment (X)	0.384	0.147	51.582	0.229	5.825	<0.001
	Positive Academic Emotions (M1)				0.252	6.403	<0.001
Academic Buoyancy Among Female Students (Y)	High School Male Teachers’ Physical Education Classroom Environment (X)	0.468	0.219	55.691	0.253	6.529	<0.001
	Positive Academic Emotions (M1)				0.218	5.612	<0.001
	Learning Engagement (M2)				0.169	4.309	<0.001

“Overall Fit Index” includes *R* (correlation), *R*^2^ (variance explained), *F* (model significance test). “Significance of Regression Coefficients” has *β* (effect size), *t* (individual predictor test), *p* (*p* < 0.05 indicates significant association).

The analytical syntax for the PROCESS macro and the complete output (including all direct and indirect effect estimates, standard errors, and confidence intervals) are provided as and are also available from the corresponding author upon request.

### 4.5 Chain mediating effect of positive academic emotions and learning engagement

Positive academic emotions and learning engagement showed a chain mediating role between high school male teachers’ physical education classroom environment and female students’ academic buoyancy. In the proposed model, physical education classroom environment was positively associated with positive academic emotions, which in turn was positively associated with learning engagement, and learning engagement was positively associated with female students’ academic buoyancy. This chain mediating pathway indicates that physical education classroom environment shows not only a direct positive association with female students’ academic buoyancy but also indirect associations through psychological processes (positive academic emotions and learning engagement). The data results are shown in Table 6, and the model diagram is presented in Figure 2.

**Table 6:** Chain mediation effects table.

Effect type	Paths	Effect estimate	Standard error	*LLCL*	*ULCL*	Proportion of total effect
Total Effect		0.3626				100%
Direct Effect	Direct Path	0.2525	0.0424	0.1937	0.3604	69.60%
Total Indirect Effect		0.1101	0.0217	0.0711	0.1551	30.40%
Indirect Effect	Path 1: X → M1 → Y	0.0599	0.0170	0.0308	0.0963	16.50%
	Path 2: X → M2 → Y	0.0386	0.0128	0.0167	0.0653	10.70%
	Path 3: X → M1 → M2 → Y	0.0116	0.0041	0.0049	0.0209	3.20%

Table shows chain mediation effects. Columns: effect types, paths, effect size, variability, significance (via LLCL/ULCL), and ratio of total effect.

**Figure 2:**
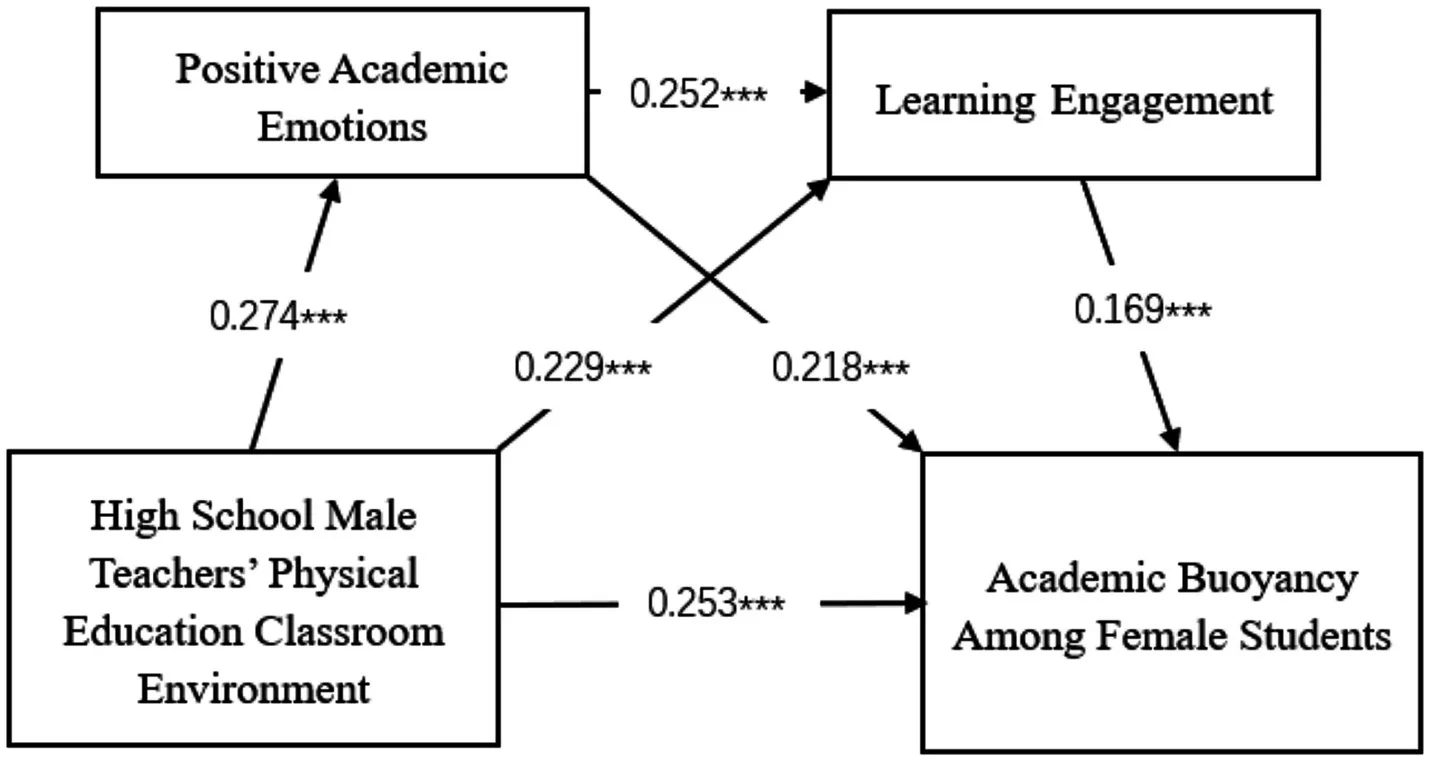
Chain mediation model diagram. The values in this figure were generated from the *β* and *p* in Table 5. In this figure, *p*-values less than 0.001 are represented by three stars, indicating high significance; *p*-values less than 0.01 are shown as two stars, denoting moderate significance; and *p*-values less than 0.05 are marked by one star, indicating significance.

## 5 Discussion

Before interpreting the findings, it is important to emphasize that the cross-sectional design of this study precludes any causal inference. All reported associations, direct and indirect, should be understood as correlational evidence that is consistent with the proposed theoretical model, rather than as confirmation of causal sequencing.

### 5.1 Discussion on differences between sample groups

The independent samples *t*-test showed (see Table 2) that there were no significant differences (*p* > 0.05) between female students with daily exercise habits (*n* = 346, 57.6%) and those without exercise habits (*n* = 255, 42.4%) in physical education classroom environment perception, academic buoyancy, positive academic emotions, and learning engagement. This finding deviates from conventional expectations.

Research indicates that female students perceive a performance-oriented classroom atmosphere less positively than male students, suggesting that competitive physical education environments may disproportionately alienate female participants (Marcelino, 2026). In the present study, the classroom context---in which all participants were taught by male teachers---may emphasize competition and public performance to varying degrees, which could lead some female students to experience body image pressure and social evaluation concerns, thereby attenuating the individual differences brought by exercise habits (Jee, 2025). However, because our study did not include a female-teacher comparison group, we cannot determine whether these patterns are specific to male-teacher-led classrooms. Moreover, as these variables (e.g., body image pressure, social evaluation concerns) were not directly measured in the present study, this interpretation remains speculative and requires direct empirical verification in future research. Furthermore, studies on adolescent females’ physical activity have found that regular exercise may positively or non-significantly affect academic performance, but excessive exercise aimed at weight control may have negative effects (Martin et al., 2017). This suggests that the relationship between exercise habits and academic-related variables is moderated by exercise motivation and contextual factors, which may mask the main effect of exercise habits in the specific male teacher classroom context of this study.

Further analysis suggests that female students with exercise habits may have accumulated higher sports confidence and physical fitness outside class. However, in a classroom environment that emphasizes competition and public performance—characteristics that may be more salient in some male-teacher-led classes—some female students might restrain their active participation. This possibility, while speculative, warrants further investigation with designs that include direct measures of classroom climate and teacher-student interaction patterns, as well as comparisons across different teacher gender conditions. In contrast, female students without exercise habits may achieve similar classroom experiences through compensatory efforts (e.g., following instructions more conscientiously) or seeking peer collaboration. However, as these compensatory behaviors were not assessed in the present study, this interpretation remains speculative and warrants empirical investigation. Relevant analyses indicate that gender role expectations significantly moderate the formation of female students’ academic buoyancy (Huang and Kou, 2025). While this finding aligns with our speculative interpretation that contextual factors may attenuate the association of exercise habits with the study variables, gender role expectations were not directly measured in the present study. Future research should include direct measures of gender role expectations to examine whether they indeed moderate the observed associations. The “happy hormone” effect induced by exercise has been shown to help relieve stress (Oliver and Hyde, 1993); however, whether this effect is counteracted by anxiety in a competitive classroom remains speculative and requires further investigation with direct measures of both anxiety and classroom climate. Therefore, simply grouping individuals by “whether they have exercise habits” may be insufficient to predict female students’ psychological and behavioral performance in specific classroom contexts. Future research should focus on more refined indicators such as exercise type, intensity, and motivation.

The one-way ANOVA results showed (see Table 3) that there were no statistically significant differences (*p* > 0.05) among female students in grades one, two, and three across all variables. This finding also deviates from conventional expectations.

Relevant research on Chinese adolescents aged 12–16 has found that Chinese students’ level of academic buoyancy is significantly higher than that of their peers in North America and the United Kingdom, indicating a certain stability of academic buoyancy across cultural contexts (Liu, 2025). Further analyses confirm that academic buoyancy, as the ability to cope with daily academic setbacks, exhibits cross-time stability during middle school, with its development being more influenced by early educational experiences and personality traits (Castillo-Jiménez et al., 2022). At the same time, exercise promotes the release of endorphins and dopamine, producing a “happy hormone” effect that alleviates stress; some senior female students have reported that physical education class is a valuable “stress-relief moment,” becoming a rare opportunity for relaxation when academic pressure is high (Ma et al., 2024). Thus, the stress-relief function of physical education classes may offset the negative impact of academic pressure on positive psychological experiences in the senior year, while female students’ rapid adaptation to the classroom environment may allow their psychological experiences to enter a stable plateau phase soon after entering high school.

Furthermore, Chinese high school physical education curricula often adopt modular teaching. Although the curriculum content progresses across grades, the overall structure remains similar, and most male physical education teachers teach across different grades, resulting in high consistency in classroom atmosphere and teaching style over the 3 years (Nowak et al., 2024). Meanwhile, as grade level increases, female students develop increasingly mature coping strategies for potential gender stereotypes in physical education classrooms (e.g., “certain sports are not suitable for girls”). This psychological resilience enables them to maintain relatively stable positive experiences even during high-stress periods. Notably, in this study, second-year female students showed a slightly lower mean value for positive academic emotions (3.616) than first-year (3.749) and third-year (3.772) students. However, this difference did not reach statistical significance (*F* = 1.614, *p* = 0.200), and given the cross-sectional nature of the data, this descriptive pattern should be interpreted with caution. Future research could adopt longitudinal designs to examine whether any developmental trends in academic emotions exist across grade levels.

### 5.2 Discussion on the direct association

The present study found that high school male teachers’ physical education classroom environment was significantly and positively associated with female students’ academic buoyancy (*β* = 0.253, *p* < 0.001), with the direct association accounting for 69.60% of the total effect. Hypothesis H1 was supported. This result indicates that a supportive physical education classroom environment dominated by male teachers is significantly associated with higher levels of female students’ ability to cope with daily academic setbacks.

The significant positive direct association between physical education classroom environment and academic buoyancy can be understood from two perspectives: resource integration and psychological empowerment. On the one hand, a supportive classroom environment provides students with emotional support, clear rules, and positive feedback; these external resources can directly strengthen students’ confidence and coping strategies when facing academic difficulties (Astin, 1965). Some physical education teachers—regardless of gender—may emphasize clear decision-making, rule enforcement, and problem-solving. To the extent that such teaching characteristics are present in the classroom, they may be positively associated with students’ handling of academic setbacks. However, because our study did not include a female-teacher comparison group, we cannot attribute these observations specifically to teacher gender. On the other hand, successful experiences in physical education classes (e.g., mastering a skill, completing a challenge) directly enhance students’ self-efficacy, which is a core antecedent of academic buoyancy (Weißenfels et al., 2022). When teachers create a classroom atmosphere characterized by fairness, respect, and moderate challenge, female students are more likely to gain a sense of competence, and this sense of competence may generalize to the academic domain, enabling them to exhibit greater resilience when facing daily setbacks such as exam failures and homework pressure. In the present study, all participating teachers were male; however, whether similar associations would be observed in classrooms led by female teachers remains an open question for future research.

Notably, the proportion of the direct association in this study (69.60%) is much higher than the sum of the subsequent mediating pathways (30.40%), indicating that the association between physical education classroom environment and academic buoyancy is primarily reflected in the direct path rather than entirely through emotional and behavioral mediators. This may be related to the embodied cognition nature of physical education itself: the immediate physiological arousal and psychological regulatory effects induced by physical activity may bypass higher-order cognitive processing and directly affect individuals’ stress-coping capacity (Frikha et al., 2024). Furthermore, an authoritative yet supportive teaching style, characterized by clear expectations and consistent feedback, may provide students with a sense of predictability and security in the classroom, which may in turn be positively associated with a stable mindset when facing academic challenges. Future research with teacher-gender comparisons is needed to examine whether such effects differ by teacher gender (Pöysä et al., 2019).

### 5.3 Discussion on the mediating role of positive academic emotions

The results of this study indicate that positive academic emotions play a partial mediating role between high school male teachers’ physical education classroom environment and female students’ academic buoyancy (indirect effect value = 0.0599, accounting for 16.50% of the total effect, 95% *CI* [0.0308, 0.0963]), supporting Hypothesis H2.

This finding is highly consistent with the control-value theory of achievement emotions. According to this theory, environmental factors (e.g., classroom quality, teacher-student interaction) indirectly shape students’ academic emotional experiences by influencing their appraisals of control and value regarding learning activities; once academic emotions are formed, they further affect learning behaviors and outcomes (Sajjad et al., 2023). In a physical education classroom dominated by male teachers, when teachers provide clear instructions, fair evaluations, moderate challenges, and positive feedback, female students’ sense of control over classroom activities is enhanced, and their recognition of the value of physical education learning also increases, making them more likely to experience high-arousal positive emotions such as pride, joy, and hope. In this study, physical education classroom environment was significantly and positively associated with positive academic emotions (*β* = 0.274, *p* < 0.001), providing empirical support for this theoretical pathway.

Further analysis suggests that positive academic emotions, serving as a key bridge connecting classroom environment and academic buoyancy, may relate to academic buoyancy through multiple pathways. From the perspective of psychological resources, positive emotions expand individuals’ cognitive resources and enhance psychological resilience, enabling students to recover more quickly from daily academic difficulties such as exam failures and homework pressure, a process that is positively associated with academic buoyancy (Xiao and Liu, 2025). Existing research has found that teachers’ interpersonal support behaviors are significantly and positively associated with students’ positive achievement emotions, which in turn are positively associated with engagement in physical education classes (Salter et al., 2025). The present study extends this logical chain to the broader domain of academic resilience (academic buoyancy), demonstrating the broader relevance of positive academic emotions in this context. Similarly, within a specific classroom context (where students are taught by male teachers) and with a specific outcome variable (academic buoyancy), this study provides further evidence for the role of achievement emotions as a mediator between environmental factors and student outcomes, adding new empirical evidence for the application of control-value theory in the field of physical education.

Notably, this study focuses on female student populations in classrooms led by male teachers. Female students may be particularly sensitive to teachers’ emotional expressions, evaluation styles, and interaction patterns (Ullrich et al., 2024). However, because the present study did not include a female-teacher comparison group, the extent to which these patterns are specifically attributable to teacher gender—rather than to other teacher characteristics such as teaching style or classroom management approach—cannot be determined. When teachers’ classroom behaviors satisfy female students’ basic psychological needs (e.g., competence, relatedness), the effect of eliciting positive emotional experiences may contribute to positive academic outcomes. This finding has important implications for optimizing physical education teachers’ instructional practices. However, because our study did not include a female-teacher comparison group, we cannot determine whether these patterns are specific to male teachers.

### 5.4 Discussion on the mediating role of learning engagement

The present study found that learning engagement also plays a partial mediating role between high school male teachers’ physical education classroom environment and female students’ academic buoyancy (indirect effect value = 0.0386, accounting for 10.70% of the total effect, 95% *CI* [0.0167, 0.0653]), supporting Hypothesis H3.

Learning engagement is a comprehensive state of behavioral engagement, emotional engagement, and cognitive engagement that individuals exhibit in learning activities; the three dimensions are interrelated and jointly influence learning outcomes and psychological adaptation (Li and Zeng, 2025). In this study, physical education classroom environment was significantly and positively associated with learning engagement (*β* = 0.229, *p* < 0.001), and learning engagement in turn was positively associated with female students’ academic buoyancy (*β* = 0.169, *p* < 0.001), forming a pathway of “environment → engagement → capability.” This finding is consistent with the core tenet of conservation of resources theory: a supportive external environment provides individuals with sufficient psychological and behavioral resources, prompting them to participate in learning activities with higher levels of engagement, and sustained learning engagement in turn helps individuals accumulate psychological capital to cope with adversity, which is positively associated with resilience to setbacks (Gao et al., 2024). While the reverse direction—that students with higher academic buoyancy may exhibit greater learning engagement—is theoretically plausible, the present study adopted the direction from learning engagement to academic buoyancy based on conservation of resources theory, which posits that sustained behavioral investment in learning activities builds psychological resources that enhance resilience to setbacks. However, given the cross-sectional nature of our data, we acknowledge that the directionality of this association cannot be empirically confirmed. Future research employing longitudinal designs is needed to examine the reciprocal relationship between learning engagement and academic buoyancy more definitively, and to determine whether the direction may vary across different educational contexts or student populations.

Specifically, in a physical education classroom dominated by male teachers, when the classroom environment possesses supportive characteristics (e.g., clear rules, fair evaluations, positive teacher-student interaction), female students tend to demonstrate greater effort, positive affect, and use of cognitive strategies. This multidimensional learning engagement not only improves learning outcomes in the physical education classroom but, more importantly, the successful experiences, skill gains, and peer recognition acquired through engagement gradually internalize into students’ self-efficacy and psychological resilience. When students encounter daily academic setbacks such as exam failures or homework pressure, this psychological capital accumulated from physical education classroom engagement can transfer to academic contexts, helping students adjust their mindset more quickly and adopt coping strategies, which may be positively associated with academic buoyancy (Simón-Chico et al., 2023).

Notably, the mediating effect of learning engagement accounts for 10.70% of the total effect, which is lower than the mediating effect of positive academic emotions (16.50%). This may suggest that in male teacher-dominated classrooms, the transmission effect of emotional experience is more direct and stronger than that of behavioral engagement, or that learning engagement may partially depend on the prior elicitation of positive emotions (which also provides evidence for the existence of the chain mediation) (Ge, 2025). Furthermore, some studies have found a complex nonlinear relationship between teachers’ classroom behaviors and students’ learning engagement in physical education; overemphasizing competition and external evaluation may instead inhibit some students’ engagement levels (Milton et al., 2025). In the present study, the observed mediating role of learning engagement suggests that teachers who balance moderate challenge with emotional support may foster higher student engagement. Future research should examine whether the relationship between teaching behaviors and learning engagement varies by teacher gender.

### 5.5 Discussion on the chain mediating roles

The core finding of this study is that positive academic emotions and learning engagement constitute a significant chain mediating pathway between high school male teachers’ physical education classroom environment and female students’ academic buoyancy (indirect effect value = 0.0116, accounting for 3.20% of the total effect, 95% *CI* [0.0049, 0.0209]), supporting Hypothesis H4. This result suggests a sequential pathway of “environment → emotion → behavior → capability.”

At the theoretical level, this chain mediating pathway is consistent with and provides empirical support for two classic theoretical frameworks. First, control-value theory posits that classroom environment induces specific academic emotions by influencing students’ appraisals of control and value regarding learning activities (Lazarides and Raufelder, 2021). The present study found that a supportive classroom environment in which students are taught by male teachers was significantly and positively associated with female students’ positive academic emotions (*β* = 0.274, *p* < 0.001), laying the emotional foundation for the chain pathway. Second, broaden-and-build theory emphasizes that positive emotions broaden individuals’ momentary thought-action repertoires, elicit exploratory behaviors, and subsequently build enduring psychological resources (Fredrickson, 2001). In this study, positive academic emotions further were positively associated with learning engagement (*β* = 0.252, *p* < 0.001), and learning engagement in turn was positively associated with academic buoyancy (*β* = 0.169, *p* < 0.001), which is consistent with the logical chain of “emotion broadening → behavior building → capability accumulation.”

It is worth noting that although the proportion of the chain mediating effect relative to the total effect is relatively small (3.20%), it represents a unique indirect pathway and cannot be simply equated to the sum of two independent mediating effects. The existence of this pathway suggests that the positive association between physical education classroom environment and female students’ academic buoyancy may be reflected not only through a single emotional pathway or a single behavioral pathway, but also through the synergistic transmission of emotion and behavior, producing an additive effect. In physical education classrooms, if teachers focus only on eliciting students’ positive emotions without guiding their transformation into sustained learning engagement, or if they emphasize only behavioral participation while neglecting emotional experience, the overall positive association with academic buoyancy may be less pronounced. This pattern may be particularly relevant in classroom contexts with strong performance or competitive orientations.

### 5.6 Practical implications and suggestions

Based on the empirical findings of this study, the following suggestions are proposed for the teaching practice of physical education teachers. While all participating teachers in this study were male, the observed associations are likely related to teaching characteristics (e.g., clarity, support, feedback) rather than to teacher gender per se. These suggestions should therefore be interpreted as general pedagogical considerations, subject to future comparative research.

First, attend to classroom environment quality given its strong direct association with academic buoyancy. The direct association accounted for 69.60% of the total effect, suggesting that a supportive classroom environment is strongly associated with female students’ academic buoyancy. Teachers may consider focusing on creating a fair, respectful, and rule-clear classroom atmosphere, avoiding excessive competition or public evaluation, and increasing cooperative activities and positive feedback.

Second, support positive academic emotions given their mediating role in the association. The mediating role of positive academic emotions accounted for 16.50% of the total effect, suggesting that teachers may benefit from attending to female students’ emotional experiences. By setting moderately challenging tasks, providing choices, and offering timely encouragement, teachers could enhance students’ sense of control and value, thereby potentially eliciting positive emotions such as pride and joy.

Third, encourage learning engagement given its mediating role in the association. The mediating role of learning engagement accounted for 10.70% of the total effect, indicating that teachers might design instructional activities that continuously attract student participation, such as group tasks, gamified practice, clear goals and feedback, to promote female students’ behavioral, emotional, and cognitive engagement.

Fourth, consider the sequential interplay of emotion and engagement given their chain mediating roles. Although the chain mediating role accounted for only 3.20% of the total effect, it suggests a synergistic pathway between emotion and behavior. Teachers could first arouse positive emotions through warm-up activities, then sustain learning engagement through challenging tasks, and finally reinforce successful experiences, potentially forming a progressive cycle of emotional arousal, behavior activation, and capability consolidation.

Fifth, go beyond differences in grade level and exercise habits, focusing on classroom quality. This study found no significant differences across grades or between exercise habit groups in any of the variables, suggesting that teachers need not assume that senior grade students or those without exercise habits are naturally disadvantaged. Instead, they could benefit all students through continuous optimization of the classroom environment.

Finally, it is important to situate these practical suggestions within a broader ecological perspective. Recent systematic reviews have highlighted that adolescents’ physical activity and sedentary behaviors are influenced by interacting factors across multiple levels—individual, interpersonal, institutional, community, and policy environments (Ciaccioni et al., 2026; Kolovelonis et al., 2024). While the present study focused on classroom environment, emotions, and engagement as proximal school-based factors, these should not be interpreted as the sole drivers of female students’ academic buoyancy. Rather, classroom-level factors interact with students’ individual characteristics (e.g., motivation, self-efficacy), interpersonal relationships (e.g., peer support, family influences), and broader school policies (e.g., physical education curriculum requirements, extracurricular opportunities). Future interventions aiming to enhance female students’ physical education experiences and academic outcomes may benefit from a multicomponent, multilevel approach that addresses determinants at multiple ecological levels, rather than focusing exclusively on classroom climate modification.

### 5.7 Limitations

This study has the following limitations, which future research may address.

First, the cross-sectional design cannot infer causal relationships. Although we have framed our findings in terms of associations and indirect pathways, the data remain correlational. Future research should adopt longitudinal or experimental designs to verify the directionality of the proposed mediating pathways.

Second, the sample was limited to female high school students from Northeast China, which restricts the generalizability of the findings to other regions, cultural contexts, or student populations. Future research should expand to other regions, school types, and include comparisons between male and female students.

Third, variable measurement relied exclusively on self-report questionnaires, which may introduce social desirability bias and shared method variance. Although we conducted multiple tests for common method bias and found no severe evidence, self-report measures remain inherently limited. Future research could incorporate multi-source data such as classroom observations, teacher ratings, peer assessments, or physiological indicators to provide a more comprehensive and objective evaluation.

Fourth, potential moderating variables (e.g., gender role beliefs, body image, self-efficacy) were not examined. Future research could include such variables in the model to explore the boundary conditions under which the observed associations hold or vary.

Fifth, the specific teaching behavior dimensions of male teachers were not distinguished. The present study treated the classroom environment as a global construct. Future research should refine the sub-dimensions of classroom environment (e.g., teacher support, task climate, performance climate) to identify the most salient facilitative factors for female students.

Sixth, the study did not include a comparison group of female physical education teachers. Consequently, we cannot determine whether the observed associations are specifically attributable to teacher gender or to other teaching characteristics (e.g., teaching style, classroom management approach, years of experience) that may covary with gender. Future research should include both male and female teacher conditions to disentangle gender-specific effects from broader teaching-quality effects.

Seventh, participants in this study were nested within schools and classes, which may violate the independence assumption of ordinary least squares regression. Although preliminary intraclass correlation coefficient calculations indicated minimal clustering effects at both school (ICC = 0.031) and class (ICC = 0.042) levels, future research should adopt multilevel modeling approaches to more rigorously account for the nested data structure. Given the minimal ICC values, we did not employ cluster-robust standard errors or multilevel modeling in the primary analysis. However, we acknowledge that even small ICCs can, in some cases, affect standard error estimates; future research with larger samples across more schools may consider applying cluster-robust standard errors as a sensitivity check.

Eighth, the study did not control for other potential sources of academic buoyancy beyond the classroom environment, such as family support, peer relationships, and individual personality traits. Additionally, the proposed model did not account for other important factors that may influence academic buoyancy, including students’ prior academic achievement, socioeconomic status, prior sports experience, attitudes toward physical education, or school- and teacher-related characteristics (e.g., school climate, teacher experience, teaching style). Future research could include these covariates to provide a more complete understanding of the determinants of female students’ academic buoyancy and to isolate the unique contribution of the classroom environment.

Ninth, although we conducted confirmatory factor analyses to verify the factor structures of the classroom environment and learning engagement scales, the high Cronbach’s alpha coefficients (*α* = 0.985 and 0.981, respectively) may suggest some degree of item redundancy. While our CFAs confirmed the factor structures and did not indicate problematic redundancy, future research could consider shortened versions of these scales to reduce participant burden while maintaining psychometric adequacy.

## 6 Conclusion

This study examined the associations of high school male teachers’ physical education classroom environment with female students’ academic buoyancy, with a focus on examining the chain mediating role of positive academic emotions and learning engagement. The results showed that physical education classroom environment was not only directly and positively associated with female students’ academic buoyancy but also showed indirect associations through the independent mediating roles of positive academic emotions and learning engagement, as well as through their chain mediating roles. The chain mediating pathway suggested a sequential indirect pathway of “environment → emotion → behavior → capability,” indicating that emotional experience and behavioral engagement may have a synergistic role in the observed associations in classroom contexts where students are taught by male teachers. This study contributes to the academic buoyancy literature by extending its application to the field of physical education and focusing on the specific male teacher–female student classroom dyad. Future research should adopt longitudinal designs, expand the sample range, and include moderating variables to further examine the causal direction and generalizability of these associations.

## Data Availability

The datasets presented in this study can be found in online repositories. The names of the repository/repositories and accession number(s) can be found: https://pan.baidu.com/s/1I1rI0P-YLMmfDLu9hTvoKg.
